# Correlates of Long-Term Survival of Patients with pN+ Esophageal Squamous Cell Carcinoma after Esophagectomy

**DOI:** 10.1155/2021/6675691

**Published:** 2021-02-17

**Authors:** Ming He, Zhan Qi, Rong Qiu, Yuanping Hu, Juan Li, Yuekao Li, Yuxiang Wang

**Affiliations:** ^1^Department of Thoracic Surgery, Fourth Hospital of Hebei Medical University, Shijiazhuang 50011, China; ^2^Department of Radiation Oncology, Fourth Hospital of Hebei Medical University, Shijiazhuang 50011, China; ^3^Department of CT/MRI, Fourth Hospital of Hebei Medical University, Shijiazhuang 50011, China

## Abstract

Esophageal squamous cell carcinoma (ESCC) is the most common pathological type of esophageal cancer in China. Patients with ESCC have poor long-term survival, especially those with lymphatic metastasis (pN + ESCC). In this retrospective study, we evaluated the correlates of long-term survival time of patients with pN + ESCC. A total of 453 patients with pN + ESCC who underwent surgical R0 resection between Jan 2008 and Sep 2011 were enrolled. The follow-up ended on December 2019. The clinical, pathological, inflammation-related factors and general survival data of these patients were analyzed using SPSS 22.0 software. The 1-, 3-, and 5-year overall survival (OS) rates were 73.7%, 34.6%, and 25.6%, respectively; the 1-, 3-, and 5-year disease-free survival (DFS) rates were 45.0%, 26.3%, and 20.4%, respectively. The median OS and DFS were 23 and 14 months, respectively. On multivariate analyses, gender, site of lesion, number of dissected lymph nodes, stage pTNM, adjuvant therapy, and neutrophil lymphocyte ratio were independent predictors of OS. Site of lesion, stage pTNM, and adjuvant therapy were independent predictors of DFS. Recursive partitioning analysis (RPA) scores of each patient were calculated based on the independent predictors of OS, and the patients were divided into 3 classes: low-risk, medium-risk, and high-risk. The OS, DFS, and local recurrence-free survival were significantly different among these three RPA classes (*P* < 0.001). Several factors showed an independent association with long-term postoperative survival of pN + ESCC patients after radical surgery. RPA scores can potentially be used to predict the prognosis of ESCC.

## 1. Introduction

Esophageal cancer (EC) is the fifth most commonly occurring cancer and the fourth leading cause of cancer-related deaths in China [1]. Esophageal squamous cell carcinoma (ESCC) is the most common pathological type of EC in China; in contrast, esophageal adenocarcinoma is the predominant type in the Western World [2]. The National Comprehensive Cancer Network (NCCN) guidelines recommend neoadjuvant chemoradiotherapy for EC patients [3]. Surgery is the primary treatment for ESCC in China as it provides a chance of cure. However, the five-year survival rates of Chinese patients with ESCC are not satisfactory. The NCCN guidelines recommend only regular follow-up for EC patients who have undergone radical surgery [3]. The difference between the therapeutic strategies employed in China and other countries may influence the general outcomes of ESCC.

A large number of patients experience recurrence after surgery, especially those with pathologic positive lymph node metastasis (pN+) [4–11]. Several clinical, pathological, and inflammatory indices are known to impact survival of patients with local advanced ESCC [4–15]. Many studies have demonstrated the influence of neutrophil lymphocyte ratio (NLR) and the lymphocyte monocyte rate (LMR) on the general outcomes of ESCC [13–15]. In our previous studies, we identified several clinical factors related to prognosis of ESCC [6, 7]. In this study, we retrospectively evaluated the long-time survival outcomes and identified their correlates in patients with stage pT2-4N1-3M0 ESCC after resection. Comparison of our results with those reported from other countries may facilitate stratification of patients based on survival risk and help direct postoperative adjuvant therapeutic strategy for these patients.

## 2. Materials and Methods

### 2.1. Eligibility

The inclusion criteria were (1) patients who had undergone radical esophagectomy with two- or three-field lymphadenectomy between January 2009 and December 2011, (2) esophageal squamous cell carcinoma, (3) pN+ and pT2-4 (pT1N+ was eliminated due to less number of cases) and without distant metastasis, (4) no history of other tumors, (5) Karnofsky performance status ≥70, and (6) survival time at least one month after surgery. The study was approved by the Medical Ethics Committee of The Fourth Hospital of Hebei Medical University. Written informed consent was obtained from all subjects.

### 2.2. Surgery

In China, the type of R0 resection surgery depends on the tumor location. Left thoracic approach (Sweet surgery) is usually performed in patients with tumor located in the middle and lower esophagus. Right thoracic approach (Ivor-Lewis surgery) is performed in patients with tumor located in the upper esophagus. Radical surgical resection consists of transthoracic subtotal esophagectomy with abdominal and mediastinal lymphadenectomy (two-field) with or without supraclavicular lymphadenectomy (three-field surgery). A gastric tube through the posterior mediastinal route is used as a substitute for the resected esophagus to restore the continuity of the alimentary tract. Pathology and staging were performed according to the AJCC/UICC 7^th^ TNM cancer staging criteria.

### 2.3. Postoperative Adjuvant Therapy

Cisplatin combination chemotherapy was used, with either fluorouracil or paclitaxel/docetaxel; the median number of treatment cycles was 3 (range, 1–8). Radiotherapy (three-dimension conformal radiotherapy or intensity modulated radiotherapy) was initiated 4–8 weeks after surgery. The clinical target volume (CTV) was designed as follows: the upper mediastinum, supraclavicular, and lower neck area for upper thoracic ESCC; whole mediastinum with/without supraclavicular area for middle thoracic ESCC; and middle and lower mediastinum and gastric left lymphatic drainage area for lower ESCC. Since the NCCN guidelines do not contain any recommendations for postoperative radiotherapy, the total dosage of radiotherapy was 50–54 Gy administered in 25–28 fractions (1.8–2.0 Gy/fraction, 5 fractions per week) for CTV. Owing to poor tolerance of patients to concomitant postoperative chemoradiotherapy (POCRT), sequential radiotherapy and chemotherapy were administered in this study population. One hundred thirty-one patients were not administered any adjuvant treatment owing to patient's refusal, intolerance, financial constraints, or other reasons.

### 2.4. Calculation of NLR and LMR

All blood samples were collected within one week before surgery; in case of multiple routine blood examinations in the same patient, the last sample obtained before surgery was used in the subsequent analyses. The NLR was calculated by dividing the number of absolute neutrophil count (×10^9^/L) by the number of absolute lymphocyte count (×10^9^/L). The LMR was calculated by dividing the absolute lymphocyte count (×10^9^/L) by the absolute monocyte count (×10^9^/L). The cutoff level for NLR was 3.5 according to the published data [14], while that for LMR was 3.5 according to the median value.

### 2.5. Follow-Up

Follow-up ended on 1, December 2019. The follow-up schedule was every three months for two years, every six months for the next three years, and annually thereafter. Contrast-enhanced computed tomography (CT) of the neck, thorax, and upper abdomen, and routine blood and biochemistry investigations were performed at each follow-up visit. Ultrasonography of neck and upper abdomen, nuclear bone scanning, gastric endoscopy, positron emission tomography, or cytologic puncture was performed, if needed.

The long-term outcomes were determined from medical records and follow-up information. Overall survival (OS) was defined as the time from operation to death (or the last follow-up visit). Disease-free survival (DFS) was defined as the time from operation to first disease failure, including locoregional recurrence (LR), distant metastasis, and combined recurrence or death from any cause. Locoregional recurrence-free survival (LRFS) rate was measured from the date of surgery to the date of first evidence of local or regional recurrence. LR included recurrence in the primary esophageal tumor bed, anastomotic sites, and regional lymph nodes (LNs) including supraclavicular, mediastinal, and celiac axis LNs. Recurrence beyond those sites was considered distant metastasis (DM). Recurrences or metastases were documented from clinical investigation reports including CT, esophagoscopy plus biopsy, or positron emission tomography (PET)/CT.

### 2.6. Statistical Analysis

All statistical analyses were conducted using SPSS 22.0 software (IBM Corp, Armonk, NY, USA). OS and DFS were calculated via Kaplan–Meier analysis, and between-group differences assessed using the log-rank test. Multivariate analyses were performed using a Cox proportional hazard regression model. *P* values <0.05 were considered indicative of statistical significance.

## 3. Results

### 3.1. Patient Characteristics

A total of 453 patients were enrolled in this study. The clinical characteristics of the study population are summarized in Table 1. At the end of follow-up, 25 patients were lost to follow-up; therefore, the rate of follow-up was 94.5%.

### 3.2. Overall Survival

**Figure 1:**
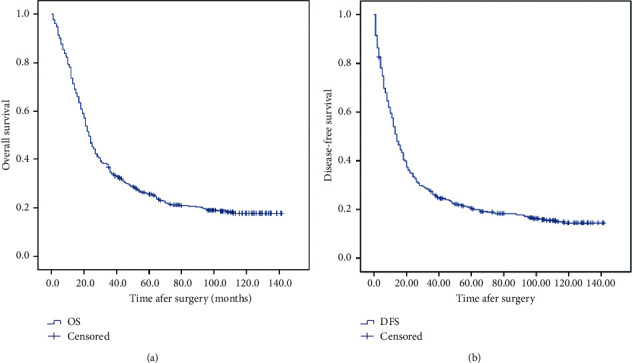
The curves of overall survival (OS) and disease-free survival (DFS) for all patients.

At the end of the follow-up, 363 patients had died, including 310 deaths due to the relapse of tumor and 53 deaths due to non-neoplastic causes. The 1-, 3-, 5-, 8-year OS and median survival time were 73.7%, 34.6%, 25.6%, 18.9%, and 23 months (20.8–25.2), respectively (Figure 1). On univariate analysis, gender, age, site of lesion, surgery (two-field/three-field), the number of dissected lymph nodes (defined as “dissected LN” below), pT, pN, pTNM, postoperative adjuvant therapy, NLR, and LMR showed a significant association with OS (*P* < 0.05). However, the tumor length and differentiation of squamous cell carcinoma (SCC) subtype showed no significant association with OS (*P* > 0.05) (Table 2).

On multivariate analyses, gender, site of lesion, dissected LN, pTNM, adjuvant therapy, and NLR were independent predictors of OS (Table 3).

### 3.3. Disease-Free Survival

The 1-, 3-, 5-, 8-year DFS and median survival time were 54.0%, 26.3%, 20.4%, 16.6%, and 14 months, respectively (Figure 1). On univariate analyses, site of lesion, pT, pN, pTNM, adjuvant therapy, and LMR showed a significant association with DFS (*P* < 0.05). However, gender, age, tumor length, surgery (two-field/three-field), dissected LN, differentiation of SCC, and NLR were not significantly related with DFS (*P* > 0.05) (Table 2). On multivariate analyses, site of lesion, pTNM, and adjuvant therapy were independent predictors of DFS (Table 4).

### 3.4. Locoregional Recurrence-Free Survival

At the end of the follow-up, 245 (54.1%) patients developed LR. The 1-, 3-, 5-, 8-year LRFS, and median LRSF time were 67.7%, 49.0%, 42.2%, 36.9%, and 24 (18.7–29.3) months, respectively. On univariate analyses, pN, pTNM, and adjuvant therapy showed a significant association with LRFS (*P* < 0.05). However, gender, age, site of lesion, length of tumor, surgery (two-field/three-field), differentiation of SCC, dissected LN, pT, LMR, and NLR were not significantly related to LRFS (*P* > 0.05) (data not shown). On multivariate analyses, site of lesion, pTNM, and adjuvant therapy were independent predictors of LRFS (Table 4).

### 3.5. Recursive Partitioning Analysis Scores

In a recent study, a recursive partitioning analysis (RPA) model was used to predict the OS of patients with ESCC [9]. Based on the six independent prognostic factors for OS, we defined risk levels as 0, 1, and 2 degrees according to the analysis of results: gender (female = 0, male = 1), site of lesion (lower or upper segment = 0, middle = 1), dissected LN (≥12 = 0, <12 = 1), pTNM (IIb + IIIa = 0, IIIb + IIIc = 1), adjuvant therapy (with = 0, without = 1), and NLR (≥3.5 = 0, <3.5 = 1).

**Figure 2:**
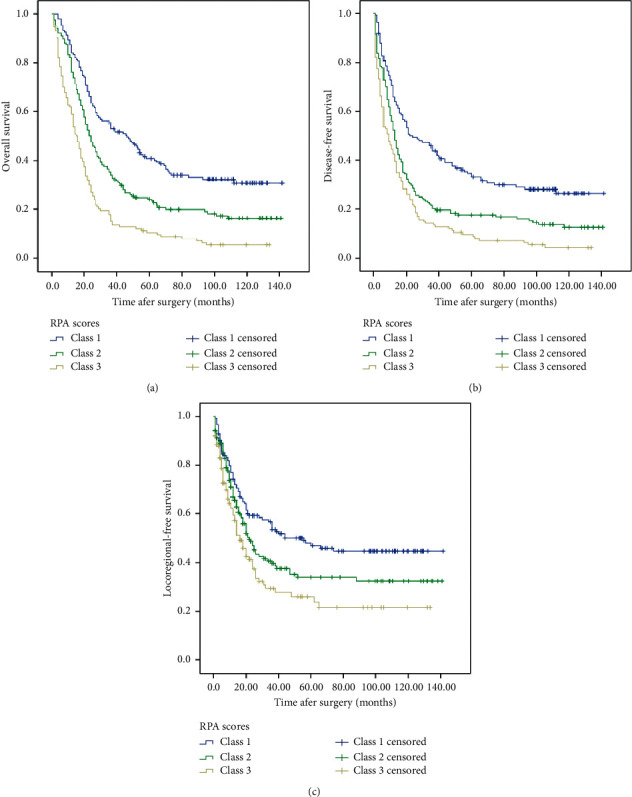
The curves of overall survival (OS), disease-free survival (DFS), and locoregional-free survival with different recursive partitioning analysis (RPA) scores (class 1 : 0–2, class 2 : 3, class 3 : 4–6).

According to our RPA score system, the patients were allocated into six groups. The number of patients with RPA scores of 0, 1, 2, 3, 4, 5, and 6 was 1, 44, 106, 168, 95, 29, and 10, respectively. We stratified the patients into 3 classes based on the RPA scores: class 1 (low-risk group: RPA score = 0–2, 151 cases); class 2 (medium-risk group: RPA score = 3, 168 cases); class 3 (high-risk group: RPA score = 4–6, 134 cases). The OS, DFS, and LRFS were significantly different among the three classes (see Figure 2). The 1-, 3-, 5-year OS and median were 84.1%, 52.9%, 40.9%, and 46 months for class 1, 76.2%, 33.9%, 24.1%, and 23 months for class 2, and 59.0%, 14.9%, 10.4%, and 15 months for class 3, respectively (*x*^2^ = 62.706, *P* < 0.001). The 1-, 3-, 5-year DFS and median were 66.0%, 44.0%, 33.1%, and 24 months for class 1, 52.4%, 20.2%, 17.7%, and 13 months for class 2, and 42.5%, 14.2%, 9.6%, and 9 months for class 3, respectively (*x*^2^ = 42.140, *P* < 0.001). The 1-, 3-, 5-year LRFS and median were 74.1%, 53.6%, 47.0%, and 56 months for class 1, 67.0%, 39.7%, 34.0%, and 22 months for class 2, and 59.3%, 29.3%, 26.0%, and 16 months for class 3, respectively (*x*^2^ = 15.951, *P* < 0.001).

## 4. Discussion

In this study, the 3-year and 5-year OS rates of patients with pN + ESCC were 34.6% and 25.6%, respectively. The 3-year and 5-year DFS rates were 26.3% and 20.4%, respectively. The median OS time was 23 months, while the median DFS time was 14 months. Lu et al. reported 22% 5-year OS rate of patients with pN + EC [16]. In our previous study, the 3-year and 5-year OS of patients after surgery for stage III EC were 34.4% and 26.7%, respectively [6]. In a study by Wu et al., the 5-year OS of patients after surgery for stage III EC was 30.4% [5]. Zou et al. reported that the 5-year OS and DFS rates of patients with stage II-III ESCC after postoperative radiotherapy (PORT) or postoperative chemoradiotherapy (POCRT) were 21.7% and 13.9%, respectively [11]. Li et al. reported 29.3% 5-year OS rate of patients with stage II-III ESCC after three-field resection [10]. All these results are consistent with those of our study. However, in a study by Zhang et al., the 5-year OS and DFS rates of patients with pN + ESCC after PORT or POCRT were 53% and 44%, respectively [17]. The survival rate was higher than that in our study; this was probably attributable to postoperative adjuvant therapy.

In our study, postoperative chemotherapy (POCT), PORT, and POCRT were found to significantly improve the OS and DFS in comparison with surgery alone (SA) for pN + ESCC patients after R0 resection, which has been summarized in our previous report [18]. Our data were consistent with previous reports, which demonstrated the survival benefit conferred by postoperative adjuvant therapy in patients with pN + ESCC [4, 9, 10, 12, 19–21].

UICC stage was another independent prognostic factor for OS and DFS in our study. In several studies, clinical stage was an independent prognostic factor for OS and DFS in patients with II-III ESCC [10, 11, 22, 23]. In other studies, pT and pN were independent prognostic factors for OS and DFS [17, 22, 24–26]. In the study by Ni et al., OS and DFS were significantly different between stages IIIb and IIIc, but not between IIIa and IIIb on univariate analysis [9]. However, Ni et al. [9] and Zhang et al. [19] found that clinical stage was not an independent predictor of OS and DFS.

In our study, primary tumor location was an independent predictor of OS and DFS. The OS and DFS were significantly lower in middle-segment than in lower-segment EC. Yu et al. [23] also demonstrated the association between tumor location and OS of patients with II-III ESCC. Other studies have also yielded similar results [6, 25]. However, in several studies, primary tumor location was not associated with OS and DFS of patients with II-III ESCC after surgery [9–11, 17, 22]. This discrepancy is likely attributable to differences with respect to the type of surgery and the study population.

The number of dissected LNs was an independent predictor of OS and DFS in this study. The OS and DFS were significantly decreased in patients with <12 dissected LNs. In a study by Dutkowski et al., the maximum increase in the sensitivity of pN classification was observed when the number of examined lymph nodes increased from 0 to 6 [27]. Examination of >12 nodes was associated with >90% sensitivity of lymph node classification. Therefore, they recommended examination of at least 12 lymph nodes for accurately defining the pN category in esophageal cancer. Hu et al. demonstrated that the UICC and AJCC recommendation for removal of at least 6 LNs is rational and should be complied with [28]. In a study by Dudash et al., EC patients in whom ≥12 nodes were removed showed better OS; the authors proposed that all centers should strive to examine at least 12 nodes to provide a quality esophagectomy [29]. Therefore, the cutoff value of dissected LNs was defined as 12 in our study. However, Altorki et al. showed significantly reduced death hazard for node-positive EC patients in whom more than 16 regional LNs were removed [30]. Rizk et al. showed that, in patients with pN + M0 EC and 1–6 positive LNs, optimum lymphadenectomy was 10 for pT1, 15 for pT2, and 29–50 for pT3/T4 [31]. Above all, dissected LNs were an important factor for survival in pN + ESCC patients.

In this study, female patients showed significantly higher OS than male patients; gender was an independent factor for OS, but not for DFS. Several other studies [17, 24, 26] have also found gender as an independent predictor of OS in ESCC patients after surgery. In the study by Li et al. [10], gender showed a significant association with OS and DFS on univariate analysis but not on multivariate analysis. However, in other studies [11, 22, 25], gender was not associated with OS in ESCC patients after surgery.

Tumor recurrence was the most frequent reason of failure for ESCC patients after surgery. In our study, adjuvant therapy was associated with LRFS. PORT and POCRT significantly increased the LRFS; however, POCT was not associated with prolonged LRFS in comparison with SA. In the study by Ni et al. [9], PORT decreased the absolute local regional recurrence rate by 20.0%. Zeng et al. [32] showed that PORT significantly reduced LR from 92.0% to 35.7%; however, DM increased from 19.0% to 75.0%. However, in the study by Li et al. [10] local recurrence rates in patients with II-III ESCC were similar among the SA, PORT, and POCRT groups (*P*=0.10); the POCRT group had significantly fewer cases of hematogenous metastasis and overall recurrence. Zhang et al. [17] found significant correlation of sequential POCRT with poor LRFS in comparison with concurrent POCRT. Site of lesion was also associated with LRFS in our study. However, Zhang et al. [17] found no association of tumor location with OS, DFS, or LRFS. We also observed an association between pTNM and LRFS in pN + ESCC patients. Sohda et al. [33] also found a significant correlation of recurrence with pT and pN.

In our study, we used the cutoff value of NLR based on the published data. Preoperative NLR was an independent predictor of OS, but not of DFS or LRFS. In the study by Feng et al. [14], ESCC patients with preoperative NLR ≥ 3.5 had significantly poorer 5-year OS rate compared to those with NLR < 3.5 (35.4% *vs* 57.7%, respectively). Gao et al. [34] found that NLR was an independent prognostic factor for OS in ESCC patients. In the study by Kosumi et al. [35], high NLR was associated with significantly shorter OS and cancer-specific survival (CSS). In the study by Duan et al. [36], the median postoperative CSS and recurrence-free survival (RFS) of patients with NLR ≤ 3 was 70 and 58 months, respectively, as against 24 and 17 months, respectively, for patients with NLR > 3. In a meta-analysis by Sun and Zhang [15], high NLR and low LMR were associated with poor OS or CSS and event-free survival of ESCC patients.

In our study, LMR was associated with OS and DFS in univariate analysis; however, LMR was not an independent predictor of OS or DFS. In the study by Gao et al. [34], LMR was an independent prognostic factor for OS in patients with ESCC. In several studies, low LMR was found to predict shorter DFS and OS in patients with ESCC [13, 37, 38]; patients with low preoperative LMR exhibited significantly worse DFS and OS than those with high preoperative LMR. Hirahara et al. [39] found a significant relationship of LMR with CSS or OS of patients with stage III EC.

Recently, nomogram and recursive partitioning analysis (RPA) scores have been used to predict the postoperative survival of patients with ESCC. Zheng et al. [24] selected five independent predictors of OS (gender, age, number of resected nodes, pT status, and pN status) to evaluate the nomogram score in ESCC patients after surgery; the 5-year OS for the four risk strata was 89.6%, 68.3%, 38.2%, and 11.5%, respectively. Deng et al. [25] found that the prognostic efficacy of the nomogram in the training and validation cohorts for patients with pT1N + /T2-4aN0-3M0 ESCC was significantly greater than that of the AJCC staging system. Duan et al. [26] used five independent prognostic variables to build the nomograms which could provide individualized risk estimates of DFS and OS for ESCC patients after POCT. Ni et al. [9] also found that RPA scores significantly predicted the risk of survival for pN + ESCC patients with PORT. Yu et al. [40] used nomogram and RPA to classify patients with IIB-III ESCC into three risk groups according to the lymph node metastatic ratio and adjuvant therapy; the 5-year OS for low-, intermediate-, and high-risk groups were 47.4%, 31.1%, and 11.7%, respectively. We classified patients with pN + ESCC after surgery into 3 classes according to the RPA scores along with six independent factors for OS; the OS, DFS, and LRFS were significantly different among the three classes. Therefore, RPA scores may help predict survival of these patients.

Some limitations of our study should be considered while interpreting the results. First, this was a retrospective single-center study. Therefore, the possibility of selection bias cannot be excluded despite the use of multivariate analysis. Second, the left thoracic approach was used for most of the patients in our study; therefore, the impact of different surgical approaches was not considered in the analysis. The number of dissected LNs was found to influence OS and DFS in our study; this was partly associated with the surgical approach. Third, the target dosages of PORT and the scheme and cycles of POCT were slightly different and were not analyzed in detail. However, our results demonstrated the survival benefit of postoperative adjuvant therapy in these patients; we intend to evaluate this issue in detail in our future study. Finally, our PRA scores were summarized from the training cohorts but not verified in the validation cohorts; this will be performed in our future study.

## 5. Conclusion

In our study, several factors were found to influence the postoperative OS, DFS, and LRFS of patients with pN + ESCC who underwent radical resection; stratification of all patients into three classes based on RPA scores significantly predicted the risk of survival, including OS, DFS, and LRFS.

## Figures and Tables

**Table 1:** Clinical characteristics.

Factors	No.
*Gender*	
Male	338
Female	115

*Age (years)*	
60 (37–83)^*∗*^	

*Site of lesion*	
Upper	40
Middle	312
Lower	101

*Length of lesion*	
6 (1–16)^*∗*^cm	

*Resected LN*	
10 (1–34)^*∗*^	

*NLR*	
2.77 (0.76–50.67)^*∗*^	

*LMR*	
3.50 (0.50–22.36)^*∗*^	

*Surgery*	
Two-field	430
Three-field	23
Factors	No.

*pT*	
pT2	57
pT3	367
pT4	29

*pN*	
pN1	294
pN2	131
pN3	28

*pTNM*	
IIb	42
IIIa	250
IIIb	108
IIIc	53

*Differentiation*	
Well-middle	331
Poor	122

*Adjuvant therapy*	
No	131
POCT	222
PORT	57
POCRT	43

^*∗*^indicates median (range); LMR: lymphocyte monocyte rate; LN: lymph node; NLR: neutrophil lymphocyte ratio; POCRT: postoperative chemoradiotherapy; POCT: postoperative chemotherapy; PORT: postoperative radiotherapy.

**Table 2:** Results of univariate analysis showing factors associated with survival of patients with pN + ESCC.

Factors	N.	OS		*P*	DFS		*P*	LRFS		*P*
		3-year	5-year		3-year	5-year		3-year	5-year	
Gender										
Male	338	34.0	23.9	0.040	25.2	18.8	0.115	40.1	34.0	0.199
Female	115	36.5	30.3		29.6	25.1		48.1	44.7	

*Age*										
≤60 years	247	37.6	28.0	0.084	26.0	21.9	0.349	40.0	35.9	0.510
>60 years	206	31.1	22.7		26.7	18.8		54.2	38.2	

*Site of lesion*										
Upper	40	47.5	30.0	0.047	38.6	25.8	0.011	48.9	45.6	0.070
Middle	312	30.0	22.3		22.4	17.3		39.8	33.1	
Lower	101	42.5	34.2		33.6	28.1		47.1	44.0	

*Length of lesion*										
<6 cm	219	37.0	26.5	0.415	28.3	22.6	0.140	42.1	35.7	0.965
≥6 cm	234	32.4	24.7		24.5	18.4		42.4	38.4	

*Resected LN*										
<12	264	31.8	23.6	0.045	23.9	18.8	0.169	41.8	37.5	0.656
≥12	189	38.6	28.2		29.8	22.7		42.5	35.7	

*Surgery*										
Two-field	430	33.5	24.4	0.071	25.3	19.3	0.202	41.8	38.9	0.322
Three-field	23	56.5	47.8		45.9	41.3		50.5	50.5	

*Differentiation*										
Well-middle	331	34.4	25.0	0.603	25.2	19.4	0.196	42.6	37.8	0.935
Poor	122	35.2	27.3		29.5	23.1		41.4	34.8	

*pT*										
pT2	57	45.6	34.4	0.007	31.6	24.9	0.003	38.6	35.4	0.121
pT3	367	34.6	25.3		27.1	20.8		44.9	38.8	
pT4	29	13.8	13.8		6.9	6.9		16.3	16.3	

*pN*										
pN1	294	42.2	32.4	<0.001	32.4	25.1	<0.001	47.6	40.6	<0.001
pN2	131	23.7	15.1		17.6	13.6		34.0	32.3	
pN3	28	7.1	3.6		3.6	3.6		10.3	10.3	

*pTNM*										
IIb	42	50.0	42.7	<0.001	35.7	29.2	<0.001	38.3	34.8	<0.001
IIIa	250	41.2	30.2		32.5	24.4		51.1	43.0	
IIIb	108	25.0	17.6		18.5	14.8		35.5	33.6	
IIIc	53	11.3	9.1		5.7	5.7		13.2	13.2	

*Adjuvant therapy*										
No	131	20.6	15.2	<0.001	15.4	13.1	<0.001	31.4	28.4	<0.001
POCT	222	36.5	28.0		27.5	20.8		39.6	33.5	
PORT	57	43.9	27.4		29.8	24.2		53.1	53.1	
POCRT	43	55.7	42.8		48.8	35.5		69.2	57.0	

*NLR*										
<3.5	314	38.5	28.7	0.002	27.8	22.2	0.070	42.3	37.5	0.473
≥3.5	139	259	18.5		23.0	16.5		42.0	35.3	

*LMR*										
≤3.5	233	30.4	21.9	0.033	22.9	18.3	0.043	40.4	34.9	0.154
>3.5	220	39.1	29.5		30.0	22.6		44.1	38.9	

LN: lymph node; POCRT: postoperative chemoradiotherapy; POCT: postoperative chemotherapy; PORT: postoperative radiotherapy; NLR: neutrophil lymphocyte ratio; LMR: lymphocyte monocyte rate.

**Table 3:** Results of multivariate analysis showing factors associated with OS of patients with pN + ESCC.

Factors	Hr (95% CI)	*P*
*Gender*		
Male	1.346 (1.053–1.722)	0.018
Female	1	

*Site of lesion*		
Upper	1.056 (0.688–1.621)	0.804
Middle	1.350 (1.037–1.758)	0.026
Lower	1	

*Resected LN*		
<12	1.258 (1.010–1.567)	0.041
≥12	1	

*pTNM*		
IIb	1	
IIIa	1.328 (0.883–1.996)	0.173
IIIb	2.055 (1.329–3.179)	0.001
IIIc	2.766 (1.719–4.450)	<0.001

*Adjuvant therapy*		
No	1	
POCT	0.603 (0.471–0.773)	<0.001
PORT	0.539 (0.379–0.765)	0.001
POCRT	0.400 (0.264–0.606)	<0.001

*NLR*		
<3.5	0.737 (0.590–0.920)	0.007
≥3.5	1	

**Table 4:** Factors associated with DFS and LRFS on multivariate COX analysis.

Factors	*DFS*		*LRFS*	
	HR (95% CI)	*P*	HR (95% CI)	*P*
*Site of lesion*				
Upper	1.045 (0.685–1.596)	0.838	1.074 (0.628–1.836)	0.795
Middle	1.373 (1.062–1.776)	0.016	1.406 (1.020–1.938)	0.038
Lower	1		1	

*pTNM*				
IIb	1		1	
IIIa	1.311 (0.884–1.944)	0.178	0.876 (0.571–1.343)	0.543
IIIb	1.873 (1.224–2.866)	0.004	1.300 (0.814–2.078)	0.272
IIIc	2.600 (1.637–4.129)	<0.001	1.870 (1.115–3.135)	0.018

*Adjuvant therapy*				
No	1		1	
POCT	0.656 (0.517–0.833)	<0.001	0.840 (0.627–1.126)	0.244
PORT	0.604 (0.426–0.857)	0.005	0.490 (0.301–0.797)	0.004
POCRT	0.388 (0.258–0.583)	<0.001	0.333 (0.190–0.584)	<0.001

## Data Availability

The datasets used and analyzed during the current study are available from the corresponding author on reasonable request.

## References

[1] Chen W., Zheng R., Baade P. D. (2016). Cancer statistics in China, 2015. *CA: A Cancer Journal for Clinicians*.

[2] Zhang H.-Z., Jin G.-F., Shen H.-B. (2012). Epidemiologic differences in esophageal cancer between Asian and Western populations. *Chinese Journal of Cancer*.

[3] Ajani J. A., D’Amico T. A., Bentrem D. J. (2019). Esophageal and esophagogastric junction cancers, version 2.2019, NCCN clinical practice guidelines in oncology. *Journal of the National Comprehensive Cancer Network*.

[4] Zhao P., Yan W., Fu H., Lin Y., Chen K.-N. (2018). Efficacy of postoperative adjuvant chemotherapy for esophageal squamous cell carcinoma: a meta-analysis. *Thoracic Cancer*.

[5] Wu C. R., Xue H. C., Zhu Z. H. (2009). Analysis of the therapeutic effect of esophagectomy with extended 2-field lymph node dissection for esophageal carcinoma. *Zhonghua Zhong Liu Za Zhi [Chinese Journal of Oncology]*.

[6] Yang Q., Wang Y. X., He M. (2016). Factors affecting on long-time survival in patients with stage III thoracic esophageal carcinoma after esophagectomy. *Zhonghua Zhong Liu Za Zhi [Chinese Journal of Oncology]*.

[7] Gao Y. H., Wang Y. X., Li J. (2017). Impact factor of postoperative prognosis of esophageal cancer patients with stage pT2N0∼1M0. *Zhonghua Zhong Liu Za Zhi [Chinese Journal of Oncology]*.

[8] Xu Y., Liu J., Du X. (2013). Prognostic impact of postoperative radiation in patients undergoing radical esophagectomy for pathologic lymph node positive esophageal cancer. *Radiation Oncology*.

[9] Ni W., Chen J., Xiao Z. (2019). Adjuvant radiotherapy for stage pN1M0 esophageal squamous cell carcinoma: results from a Chinese two‐center study. *Thoracic Cancer*.

[10] Li L., Zhao L., Lin B. (2017). Adjuvant therapeutic modalities following three-field lymph node dissection for stage II/III esophageal squamous cell carcinoma. *Journal of Cancer*.

[11] Zou B., Tu Y., Liao D. (2020). Radical esophagectomy for stage II and III thoracic esophageal squamous cell carcinoma followed by adjuvant radiotherapy with or without chemotherapy: which is more beneficial?. *Thoracic Cancer*.

[12] Huang Y., Sun Y., Peng P., Zhu S., Sun W., Zhang P. (2017). Prognostic and clinicopathologic significance of neutrophil-to-lymphocyte ratio in esophageal squamous cell carcinoma: evidence from a meta-analysis. *OncoTargets and Therapy*.

[13] Han L.-H., Jia Y.-B., Song Q.-X., Wang J.-B., Wang N.-N., Cheng Y.-F. (2015). Prognostic significance of preoperative lymphocyte-monocyte ratio in patients with resectable esophageal squamous cell carcinoma. *Asian Pacific Journal of Cancer Prevention*.

[14] Feng J.-F., Huang Y., Chen Q.-X. (2014). Preoperative platelet lymphocyte ratio (PLR) is superior to neutrophil lymphocyte ratio (NLR) as a predictive factor in patients with esophageal squamous cell carcinoma. *World Journal of Surgical Oncology*.

[15] Sun Y., Zhang L. (2018). The clinical use of pretreatment NLR, PLR, and LMR in patients with esophageal squamous cell carcinoma: evidence from a meta-analysis. *Cancer Management and Research*.

[16] Lu J.-C., Tao H., Chen Z.-Z., Qian P.-D. (2009). Prognostic factors of radiotherapy in patients with node-positive thoracic esophageal squamous cell carcinoma after radical surgery. *Diseases of the Esophagus*.

[17] Zhang Z., Xu L., Di X., Zhang C., Ge X., Sun X. (2019). A retrospective study of postoperative radiotherapy for locally advanced esophageal squamous cell carcinoma. *Annals of Palliative Medicine*.

[18] Li J., Qiu R., Hu Y. (2021). Postoperative adjuvant therapy for patients with pN+ esophageal squamous cell carcinoma. *BioMed Research International*.

[19] Zhang L., Li W., Lyu X. (2017). Adjuvant chemotherapy with paclitaxel and cisplatin in lymph node-positive thoracic esophageal squamous cell carcinoma. *Chinese Journal of Cancer Research*.

[20] Qin R. Q., Wen Y. S., Wang W. P. (2016). The role of postoperative adjuvant chemotherapy for lymph node-positive esophageal squamous cell carcinoma: a propensity score matching analysis. *Medical Oncology*.

[21] Chen J., Pan J., Zheng X. (2012). Number and location of positive nodes, postoperative radiotherapy, and survival after esophagectomy with three-field lymph node dissection for thoracic esophageal squamous cell carcinoma. *International Journal of Radiation Oncology^∗^Biology^∗^Physics*.

[22] Song T., Chen P., Fang M., Zhang X., Du D., Wu S. (2020). The role of adjuvant chemoradiotherapy over radiotherapy after R0 resection for stage II-III esophageal squamous cell carcinoma. *Cancer Management and Research*.

[23] Yu J., Ouyang W., Li Y. (2019). Value of radiotherapy in addition to esophagectomy for stage II and III thoracic esophageal squamous cell carcinoma: analysis of surveillance, epidemiology, and end results database. *Cancer Medicine*.

[24] Zheng Y., Fu S., He T., Yan Q., Di W., Wang J. (2018). Predicting prognosis in resected esophageal squamous cell carcinoma using a clinical nomogram and recursive partitioning analysis. *European Journal of Surgical Oncology*.

[25] Deng W., Zhang W., Yang J. (2019). Nomogram to predict overall survival for thoracic esophageal squamous cell carcinoma patients after radical esophagectomy. *Annals of Surgical Oncology*.

[26] Duan J., Deng T., Ying G. (2016). Prognostic nomogram for previously untreated patients with esophageal squamous cell carcinoma after esophagectomy followed by adjuvant chemotherapy. *Japanese Journal of Clinical Oncology*.

[27] Dutkowski P., Hommel G., Böttger T., Schlick T., Junginger T. (2002). How many lymph nodes are needed for an accurate pN classification in esophageal cancer? Evidence for a new threshold value. *Hepato-gastroenterology*.

[28] Hu Y., Hu C., Zhang H., Ping Y., Chen L.-Q. (2010). How does the number of resected lymph nodes influence TNM staging and prognosis for esophageal carcinoma?. *Annals of Surgical Oncology*.

[29] Dudash M. J., Slipak S., Dove J. (2019). Lymph node harvest as a measure of quality and effect on overall survival in esophageal cancer: a national cancer database assessment. *American Surgeon*.

[30] Altorki N. K., Zhou X. K., Stiles B. (2008). Total number of resected lymph nodes predicts survival in esophageal cancer. *Annals of Surgery*.

[31] Rizk N. P., Ishwaran H., Rice T. W. (2010). Optimum lymphadenectomy for esophageal cancer. *Annals of Surgery*.

[32] Zeng Y., Yu W., Liu Q. (2019). Difference in failure patterns of pT3-4N0-3M0 esophageal cancer treated by surgery vs surgery plus radiotherapy. *World Journal of Gastrointestinal Oncology*.

[33] Sohda M., Saito H., Kuriyama K. (2019). Post-esophagectomy adjuvant chemotherapy benefits esophageal cancer patients. *In Vivo*.

[34] Gao Y., Guo W., Cai S. (2019). Systemic immune-inflammation index (SII) is useful to predict survival outcomes in patients with surgically resected esophageal squamous cell carcinoma. *Journal of Cancer*.

[35] Kosumi K., Baba Y., Ishimoto T. (2016). Neutrophil/lymphocyte ratio predicts the prognosis in esophageal squamous cell carcinoma patients. *Surgery Today*.

[36] Duan H., Zhang X., Wang F. X. (2015). Prognostic role of neutrophil-lymphocyte ratio in operable esophageal squamous cell carcinoma. *World Journal of Gastroenterology*.

[37] Song Q., Wu J.-z., Wang S. (2019). Low preoperative lymphocyte to monocyte ratio serves as a worse prognostic marker in patients with esophageal squamous cell carcinoma undergoing curative tumor resection. *Journal of Cancer*.

[38] Hu G., Liu G., Ma J.-Y., Hu R.-J. (2018). Lymphocyte-to-monocyte ratio in esophageal squamous cell carcinoma prognosis. *Clinica Chimica Acta*.

[39] Hirahara N., Matsubara T., Kawahara D., Nakada S., Ishibashi S., Tajima Y. (2017). Prognostic significance of preoperative inflammatory response biomarkers in patients undergoing curative thoracoscopic esophagectomy for esophageal squamous cell carcinoma. *European Journal of Surgical Oncology*.

[40] Yu S., Zhang W., Ni W. (2016). Nomogram and recursive partitioning analysis to predict overall survival in patients with stage IIB-III thoracic esophageal squamous cell carcinoma after esophagectomy. *Oncotarget*.

